# Supplementary material for: Real-space observation of the dissociation of a transition metal complex and its concurrent energy redistribution
Source: Nat Commun. 2025 May 22;16:4767. doi: 10.1038/s41467-025-60009-8 (PMC12098838; doi:10.1038/s41467-025-60009-8)
Supplement: Supplementary file 1 — Supplementary Information [file 41467_2025_60009_MOESM1_ESM.pdf]

Supplementary notes:
Real-space observation of a transition metal complex dissociation and its concurrent energy redistribution

Aviad Schori[1], Elisa Biasin[1,2], Ambar Banerjee[3,4], Sébastien Boutet[5], Philip H. Bucksbaum[1,6], Sergio Carbajo[5], Kelly J. Gaffney[1,7], James Glownia[5], Robert Hartsock[1], Kathryn Ledbetter[1], Andreas Kaldun[1], Jason E. Koglin[5], Kristjan Kunnus[1], Thomas J. Lane[5], Mengning Liang[5], Michael P. Minitti[5], Jordan T. O'Neal[1], Robert M. Parrish[1], Frederic Poitevin[1], Jennifer M. Ruddock[8], Silke Nelson[5], Brian Stankus[8,9], Peter M. Weber[8], Thomas J.A. Wolf[1], Michael Odelius[3], Adi Natan[1*]

[1]Stanford PULSE Institute, SLAC National Accelerator Laboratory, Menlo Park, CA, USA,
[2]Physical Sciences Division, Pacific Northwest National Laboratory, Richland, WA, USA,
[3]Department of Physics, Stockholm University, Stockholm, Sweden,
[4]RISE, TCG Centres for Research and Education in Science and Technology, Kolkata, India,
[5]Linac Coherent Light Source, SLAC National Accelerator Laboratory, Menlo Park, CA, USA,
[6]Department of Physics and Applied Physics, Stanford University, Stanford, CA, USA,
[7]Department of Chemistry, Stanford University, Stanford, CA, USA,
[8]Department of Chemistry, Brown University, Providence, RI, USA,
[9]Department of Chemistry and Biochemistry, Western Connecticut State University, Danbury, CT, USA.
* Corresponding author: natan@stanford.edu

Contents

1 Data collection and initial scattering analysis **3**

2 Extrapolation of the simulated pair density dynamics **4**
2.1 Extrapolation of dissociative pairs with DART 5
2.2 Extrapolation of non-dissociative pairs 7
2.3 Pair density error estimates for extrapolation 8

3 Simulating scattering from trajectories **8**

4 Real-space inversion of scattering signals **9**

5 Kinetic model fit for first and second CO dissociation **10**

6 Estimation of the dissociation velocity and rotation frequency **11**
6.1 From simulation: 11
6.2 From experimental ΔPD: 11
6.3 Energy partition analysis of a dissociating and rotating CO Molecule 11

7 Normal mode analysis for Fe(CO)$_4$ **12**
7.1 Determining the Contribution of Modes to Experimental Observations 13

8 Estimation of the experimental instrument response function **14**

9 Laser pulse energy scan **14**

10 Supplementary Figures **16**

Suppl. Note 1.

Data collection and initial scattering analysis

The Data collection and analysis to extract the scattering difference curves is described in detail in a previous study [1]. The analysis includes a “dark” detector correction, where an average image of the Cornell–SLAC Pixel Array Detector (CSPAD) without incident X-rays is subtracted from the raw data, using a common mode correction procedure to individual ASIC elements. Then masking of unusable pixels, such as unbonded, bright, or damaged pixels was applied. The images are then corrected for scattering geometry for the case of a plane detector, and polarization of the LCLS pulses, as detailed elsewhere [2, 3]. Images are sorted using a jitter correction timing tool and averaged to form pump-probe delay bins with similar SNR. These bins, varying in size from 17 to 26 fs, contain a comparable number of scattering events. The images are then analyzed and resampled to a uniform delay resolution of 25 fs.

Obtaining the difference scattering signals requires subtraction from a laser on and laser off shot under similar X-ray pulse conditions in a way that had to account for a slowly increasing X-ray fluorescence background signal due to the accumulation of elemental Fe atoms in the scattering cell. To obtain an appropriate representative of a laser off-shot to a particular laser on-shot, we binned all laser off-shots in a given experimental run along four dimensions: their arrival lab time, the electron beam energy that correlates with the x-ray photon energy, the detector total scattering intensity, and a diode current that records the intensity (or how many photons) of the X-ray beam. The latter two parameters provide information regarding signal degradation due to the accumulation of fluorescence background. We optimized the bin sizes to achieve a comparable level of fidelity to the laser on-shots at each time delay. We selected the bin size parameters that minimized the noise variance for negative pump-probe time delays ($\tau < 0$), where we anticipate a zero mean scattering signal. This approach extends previous methods used in ultrafast scattering, which often average laser off-shots at a given event time nearest neighbor size, or average the entire subset of laser off-shots of a particular run. This way we improved the signal-to-noise ratio while considering a dynamic fluorescence background, and accounting for the bin size adjustment to the signal SNR.

To increase the fidelity of the scattering signal we used its cylindrical symmetry property by four-folding and applying a weighted averaging of the scattering image quadrants, as detailed in [1]. This allows the reduction of the effective missing pixel data due to masking or the gaps between the ASIC elements of CSPAD detector. The signal is then transformed from Cartesian pixel space and binned into (Q, θ_d) polar detector bins for further isotropic and anisotropic scattering analysis.

Assuming ultrashort laser pump and X-ray probe pulses, the differential scattering cross section from an ensemble of excited molecules, at a pump-probe delay time τ, can be expressed by [4]:

$$\Delta \frac{d\sigma}{d\Omega} \simeq 4\pi\sigma_T[\Delta S_0(Q,\tau) - P_2(\cos\theta_Q)\Delta S_2(Q,\tau)],$$

with σ_T being the classical Thomson scattering cross section from a free electron, $Q{=}\frac{4\pi}{\lambda}\sin(\frac{\theta_Q}{2})$ is the scattering vector magnitude, with the scattering angle θ_Q and wavelength λ, and $\Delta S_n(Q,\tau)$ are the diffuse scattering curves at time delay τ and anisotropy order n (n=0 for isotropic, n=2 for anisotropic), given by:

$$\Delta S_n(Q,\tau) = \sum_{b\neq a} f_a^*(Q) f_b(Q) \int_{-1}^{1} d\cos(\theta_{ab}) \int_0^\infty dR R^2 \Delta\rho_{ab}(R,\cos(\theta_{ab}),\tau) P_n(\cos(\theta_{ab})) j_n(QR),$$

the double sum is over all atom pairs, $f_a(Q)$ is the atomic form factor of the atom a, $\Delta\rho_{ab}$ is the time-dependent pair density difference of atoms a, b at time-delay τ weighted by P_n the Legendre

polynomials, with θ_{ab} the angle between the a, b-vector and the transition dipole moment, and j_n is the n-th order spherical Bessel function of the first kind.

The scattering curves are obtained from the pattern measured on the detector by applying a Legendre decomposition over the detector angle θ_d for each Q bin, as described in [1]:

$$I(Q, \theta_d) = \beta_0(Q) \sum_{n=0,2,...} \widetilde{\beta}_n(Q) P_n(\cos\theta_d)$$

With the radial detector intensity $\beta_0(Q)$ and the normalized detector anisotropy terms $\widetilde{\beta}_n(Q) = \beta_n(Q)/\beta_0(Q)$. The relation between the anisotropy curve $S_n(Q)$ and the corresponding $\beta_n(Q)$ term is given by

$$S_n(Q) = \frac{\beta_0(Q)\widetilde{\beta}_n(Q)}{\cos^n(\theta_Q)} = \frac{\beta_n(Q)}{\left(1 - \frac{Q^2}{4|k_0|^2}\right)^{n/2}}, \text{ n=0,2,...}$$

where $|k_0|$ is the length of the wave vector of the incoming X-ray beam.

The estimation of uncertainty for the scattering curves is detailed [1]. Briefly, we first calculate the experimental weighted sample variance of each detector bin $\Delta I(q, \theta_d)$ element at each time bin delay. The variance for the kth time delay difference signal $\Delta I^{(k)}(q, \theta_d)$ is obtained by:

$$Var(I^{(k)}) = \frac{1}{N^{(k)}} \sum_{m=1}^{N^{(k)}} |w(q, \theta_d)\big(\Delta I^{(k)}(q, \theta_d) - \Delta I_m^{(k)}(q, \theta_d)\big)|^2$$

where $N^{(k)}$ is the number of images recorded for that time delay bin, $w(q, \theta_d)$ is the statistical weight each element in (q, θ_d) has due to the four-folding and detector pixel binning, $\Delta I^{(k)} = I^{(k)} - \langle I^{\tau<0} \rangle$ is the weighted average intensity difference of the kth time bin, and ΔI_m expresses the mth image that belongs to the kth time bin. The measured variance is then translated to a weights vector in the Legendre fitting process that was described. The standard error obtained from the fit is calculated for each Q bin and is propagated to each anisotropy order.

To prepare for real space inversion as detailed in Section 4, we resampled the scattering signal in Q bins with $\Delta Q = 0.1208\text{Å}^{-1}$ to improve signal fidelity, where the i-th bin is: $Q_i = \frac{j_{0i}}{j_{0N}} N\Delta Q$, where $Q_{max} = N\Delta Q = 4.34\text{Å}^{-1}$, sampled on j_{0i}, the ith zero of the spherical Bessel function j_0. The measured difference scattering curves are shown in Suppl. Fig. 1.

Suppl. Note 2.

Extrapolation of the simulated pair density dynamics

The simulated trajectories in this study exhibited a range of termination times (120-640 fs) due to non-convergence, with an average termination time of 400 fs. To ensure a comprehensive analysis of all trajectory simulations and include as many trajectories as possible, we developed a method to extrapolate the nuclear dynamics of prematurely terminated trajectories to extend them to the longest observed termination time of 640 fs. This approach enables us to average trajectories up to a time delay that captures the details of the initial CO loss. We applied the extrapolation to trajectories that met the following criteria: a termination time greater than 210 fs and a period of at least 120 fs from dissociation to termination. As a result, we successfully extrapolated 103 of the initial 116 simulated trajectories as detailed below.

Given that all measured data in the scattering process are derived from the dynamics of pair densities, we categorize the 55 unique atom pairs in $Fe(CO)_5$ into two types: (i) dissociative pairs,

involving one atom from the dissociating CO and another from the remaining $Fe(CO)_4$ (18 pairs); and (ii) non-dissociative pairs, which consist solely of atoms within the intact $Fe(CO)_4$ (36 pairs), including the single CO pair distance of the dissociating CO.

To extrapolate the dynamics of the dissociative pairs, we developed a Dissociative Atomic Rotational-Translational (DART) model, detailed in Section 2.1. This model utilizes trajectory data to fit dissociation parameters such as the onset time of dissociation, dissociation velocity, and CO rotation frequency. The approach accounts for the fact that the dynamics of dissociative pair distances—specifically those greater than 6.5 Å—are predominantly influenced by dissociation motion. In contrast, non-dissociative pairs are generally associated with distances less than 6 Å, where individual pair distance variations average below 1 Å. As a result, dissociative pair distances are primarily governed by dissociation dynamics, while inaccuracies in the motion of non-dissociative pairs, due to electronic state convergence, have minimal impact on distances greater than 6.5 Å.

For non-dissociative pairs, we apply Burg's method, an autoregressive modeling technique, described in Section 2.2. This method estimates the coefficients of a time series by minimizing forward and backward prediction errors while ensuring the model's stability. The combined outcome will be the extrapolated atomic pair motions for all pairs, along with key dissociation parameters such as dissociation velocity and rotation frequency.

The approach is validated by selecting a subset of early trajectory times for extrapolation and comparing the extrapolated pair distances with the calculated pair distances at later times in the longer-lived trajectories. The accumulated average error as a function of extrapolation time is then calculated. Details of the DART model and its fitting procedure are illustrated in Suppl. Fig. 3. The results of the extrapolation and validation for a single trajectory are shown in Suppl. Fig. 4. The total pair density dynamics, averaged across all trajectories after extrapolation, along with a detailed breakdown by different types of pairs, is presented in Suppl. Fig. 5. This figure also reveals that while motion initiates from changes in Fe-C bond distances, it influences other atomic pairs that act as "spectators," as explained by the spectator effect concept. The accumulated average extrapolation error for dissociative pairs and its impact on the extrapolated pair densities are discussed in Section 2.3 and presented in Suppl. Fig. 6.

2.1 Extrapolation of dissociative pairs with DART

To simplify the mathematical description of the motion of the dissociating CO and an atom A from the remaining $Fe(CO)_4$, the motion will be described from the reference frame of the center of mass of the dissociating CO. Since a rotation of a diatomic is restricted to a plane, without loss of generality, we will have the CO motion to be on the X-Y plane, where its center of mass is at the origin, and the C and O axis is at an initial angle $\phi(0) = 0$. We shall term the new reference frame and the transformation to get to it as the DART frame and transformation. Consequently, in the DART frame post-dissociation, the CO will appear to orbit the origin. The center of mass of $Fe(CO)_4$, represented by $\boldsymbol{r_4}$, will drift at a constant velocity in a specific 3D direction predominating the motion of atom A, that is displaced $\boldsymbol{A_0}$ away, but moves along the same direction while fluctuating relative to $Fe(CO)_4$ center of mass. The time-dependent positions of all atoms in the DART model are described by:

$$\boldsymbol{C}(\tau) = -d_C \begin{bmatrix} \cos(\phi(\tau)) \\ \sin(\phi(\tau)) \\ 0 \end{bmatrix}, \qquad \boldsymbol{O}(\tau) = d_O \begin{bmatrix} \cos(\phi(\tau)) \\ \sin(\phi(\tau)) \\ 0 \end{bmatrix}, \qquad \boldsymbol{A}(\tau) = \boldsymbol{r_4}(\tau, \mathbf{p}) + \boldsymbol{A_0} + \boldsymbol{\delta A}(\tau),$$

Where $\boldsymbol{C}$, $\boldsymbol{O}$ and $\boldsymbol{A}$ are the modeled vector positions of the atoms in the DART frame, d_C and d_O the distances of atoms C and O from the origin, given by: $d_i = \frac{m_i}{m_C + m_O} d_{CO}$, with m_i their

respective masses, and d_{CO} the CO bond distance. At the time of dissociation $\tau = \tau_d$, described by the Heaviside function Θ, the dissociated CO starts to rotate around the origin described by $\phi(\tau)=\omega_{CO}(\tau - \tau_d) \cdot \Theta(\tau - \tau_d)$, at a frequency ω_{CO}. At the same time, the center of mass of the remaining $Fe(CO)_4$ is modeled by :

$$\mathbf{r_4}(\tau, \mathbf{p}) = \mathbf{R}_0 + \mathbf{R_4} \cdot \left[v_d \cdot \left(\frac{1}{2} + \frac{1}{2} \tanh\left(\alpha \cdot (\tau - \tau_d)\right) \right) \cdot (\tau - \tau_d) \right]$$

that is initially positioned at $\boldsymbol{R_0}$, starts to drift in the direction $\boldsymbol{R_4}$ with the dissociation velocity v_d, at an onset rate α (where we used for short the DART model parameters $\mathbf{p} = [\tau_d, v_d, \alpha, \boldsymbol{R_0}, \boldsymbol{R_4}]$. The 3D description of atom A relative to the modeled $Fe(CO)_4$ center of mass $\mathbf{r_4}$ is modeled by its displacement position $\boldsymbol{A_0}$ and relative motion $\boldsymbol{\delta A}(\tau)$.

To extrapolate the pair distances using this model requires validating the transformation to the new coordinates, and obtaining the parameters: τ_d, ω_{CO}, v_d, $\boldsymbol{R_0}$, $\boldsymbol{R_4}$, $\boldsymbol{A_0}$, and $\boldsymbol{\delta A}$. Below we detail the steps to achieve that.

DART transformation: We first obtain an estimate to τ_d^{est} and velocity v_d^{est} by obtaining from the XYZ trajectory information $\Delta\sigma(\tau) = \|\boldsymbol{\sigma_{CO}}(\tau) - \boldsymbol{\sigma_4}(\tau)\|$, the time-dependent distance between $\boldsymbol{\sigma_{CO}}(\tau)$ the center of mass of the CO, and $\boldsymbol{\sigma_4}(\tau)$ the center of mass of $Fe(CO)_4$, and fit it with $\Delta\sigma(0)+v_d^{est}(\tau-\tau_d^{est}) \cdot \Theta(\tau-\tau_d^{est})$. We then use the XYZ position of $\boldsymbol{\sigma_{CO}}(\tau_d)$ to translate the coordinate system such that it is set at the origin and rotate it to set the CO positions on the X-Y plane. Once we have the rotation matrix, we use it to transform all trajectory time points, first translating by the time-dependent $\boldsymbol{\sigma_{CO}}(\tau)$, then rotating with the same rotation found for $\tau=\tau_d^{est}$. However, the rotation transformation was done using the CO positions at a single time point τ_d, and doesn't necessarily include the information regarding the plane where CO motion takes place for $\tau>\tau_d^{est}$. To validate CO motion on the X-Y plane we find the plane on which all the CO positions lie for all $\tau>\tau_d^{est}$ and rotate it to lie on the X-Y plane as needed. This is done using Singular Value Decomposition (SVD) which decomposes the set of centered CO positions into $U\Sigma V^T$. Here, V is an orthogonal matrix with columns $\mathbf{v}_1$, $\mathbf{v}_2$, and $\mathbf{v}_3$, representing the principal directions of the point cloud. The third column $\mathbf{v}_3$ corresponds to the smallest singular value and is the normal vector to the best-fit plane. To align this normal vector with the Z-axis, a rotation matrix is constructed using Rodrigues' rotation formula [5] and applied to all points, aligning the plane with the X-Y plane. Thus, $\mathbf{v}_3$ is used because it represents the direction perpendicular to the plane, capturing the least variance in the data. The results of the DART transformation are shown in Suppl. Fig. 3.

DART fit: After the DART transformation is implemented for all atomic positions and times, we can obtain the DART parameters as follows. The CO rotation frequency ω_{CO} is estimated by first taking the time derivative of the azimuthal angle θ of the rotating CO for times $\tau > \tau_d^{est}$, applying a low-pass filter to the result, and then using the median value of the filtered data as the estimate, as shown in Suppl. Fig. 3c. The CO positions are extrapolated by advancing $\phi(\tau)$ once ω_{CO} has been estimated.

To estimate the DART parameters $\mathbf{p}$, we minimize the error between the transformed XYZ data points of the $Fe(CO)_4$ center of mass $\boldsymbol{\sigma_4}^{DART}(\tau_i)$ and the model $\mathbf{r_4}(\tau, \mathbf{p})$:

$$\arg\min_{\mathbf{p}} \sum_{i=\tau_d^{est}}^{\tau_f} w_i \left\| \boldsymbol{\sigma_4}^{DART}(\tau_i) - \mathbf{r_4}(\mathbf{p}, \tau_i) \right\|^2$$

The time range extends from the estimated dissociation time point τ_d^{est} to the termination time τ_f. We apply a weight vector $w_i = \sqrt{\tau_i - \tau_d^{est}}$ to give greater emphasis to the later times, allowing the ballistic dynamics to dominate the fit and minimizing the influence of the transition to ballistic

motion. The result of such a fit is shown in Suppl. Fig. 3b. We further use the fit result $\mathbf{p}_f$, and asses $\boldsymbol{A_0}$, which is the average static displacement of atom A from $\boldsymbol{r_4}$:

$$\arg\min \sum_{i=\tau_d}^{\tau_f} w_i \left\| \boldsymbol{A}^{DART}(\tau_i) - (\mathbf{r_4}(\tau_i) - \mathbf{A_0}) \right\|^2$$

Where $\boldsymbol{A}^{DART}(\tau_i)$ represents the XYZ trajectory positions of atom A after the DART transformation. Following this fit, the model expresses the positions of $\boldsymbol{A}$ as drifting along $\boldsymbol{r_4}$ in the same direction, with a 3D displacement characterized by $\boldsymbol{A_0}$. The remaining part, $\boldsymbol{\delta A(\tau)}$, describes residual motions of atom A around the equilibrium distance from $Fe(CO)_4$ center of mass. These motions are typically <1Å and are extrapolated using Burg's method as detailed for the non-dissociative pairs in section 2.2.

The results of a typical trajectory extrapolation are shown in Suppl. Fig. 4b, where only the first 350 fs (out of 608 fs) of the trajectory is used for the DART model fit. We plot the CA and OA pair distances from the trajectory and compare them to the DART model predictions. The model excludes the vibration motion that may occur post-photoexcitation in the dissociating pairs, as its primary focus is on extrapolating the effects of dissociation translation and rotation. Instead, these motions are extrapolated using the method detailed in the next section.

2.2 Extrapolation of non-dissociative pairs

Upon examining the non-dissociative pairs, we observe that their movements are confined to distances less than 6Å, with minimal variation. We hypothesize that the motions of these pairs can be characterized by a linear combination of frequency components, which stem from their rovibrational modes, and to extrapolate their dynamics we'll apply Burg's method [6].

Briefly, this method offers a robust approach to spectral estimation by fitting an autoregressive (AR) model to a time series. The method assumes that there is a linear phase relationship between successive time samples, which in our case is composed of the non-dissociating pair distances as a function of time. The method is known for its ability to minimize prediction error and ensure forecasting stability by considering the minimization of both forward and backward prediction errors.

At a time point t, the value of the time series X_t is expressed by a linear combination of its previous values plus a noise term: $X_t = \sum_{i=1}^{p} a_i X_{t-i} + e_t$, where a_i are the coefficients of the model, p represents the order of the model, and e_t is a white noise error term. The model order p is selected by minimizing the Bayesian Information Criterion (BIC), to balance the fidelity of the model to the data with the complexity introduced by a higher number of parameters to avoid overfitting. The BIC is computed based on the final prediction error and the number of parameters $BIC = \ln(N)p + N\ln(\sigma^2)$, where $\sigma^2 = \frac{1}{N-p}\sum_{t=p+1}^{N}(\hat{X}_t - X_t)^2$ is the variance of the prediction error, and N is the number of observations.

In addition, the prediction is tested against lower and upper bounds for X_t that are set based on the physical limits for the relevant pair distance ranges for intramolecular distances. If the method does not able to extrapolate within the physically justified limits, the prediction order is iteratively decreased until it satisfies the distance bounds. The coefficients a_i are iteratively found via a modified Levinson-Durbin recursion. Initially, $a[1] = \frac{R[1]}{R[0]}$, with $R[i] = \frac{1}{N}\sum_{t=1}^{N-i} X_t X_{t+i}$ the autocorrelation coefficients at lag i. For each subsequent order k, the reflection coefficient k_k is computed,

$$k_k = \frac{-2\sum_{j=0}^{N-k-1} f_{j+k+1} b_{j+1}}{\sum_{j=0}^{N-k-1}(f_{j+k+1}^2 + b_{j+1}^2)},$$

where f and b denote the forward and backward prediction errors, respectively. Then $a[k]$ is updated by $a[j] \leftarrow a[j] - k_k \cdot a[k-j]$ for $j = 1$ to $k-1$, refining the model to minimize prediction errors. Extrapolation is obtained via $\hat{X}_{t+1} = \sum_{i=1}^{p} a_i X_{t+1-i}$, allowing predictions that extend beyond the range of the observed data.

Using Burg's method for extrapolation, we assume that the dynamics between pairs do not involve non-linear interactions, we justify this assumption as we used it to extrapolate dynamics over short timescales. Specifically, it was applied for durations on the order of a typical bending mode period of $Fe(CO)_4$ or less. Within such short intervals, the linear phase relationships between samples predominantly influence the signal behavior.

2.3 Pair density error estimates for extrapolation

In Suppl. Fig. 6 we present the estimated error in pair density when extrapolating using the DART model. The pair-distance error is calculated as a function of different trajectory termination times, considering that all trajectories used in the analysis are uniformly distributed in termination times. For the ground truth, we use a trajectory with a termination time greater than 600 fs and then compute the weighted pair-distance error when applying the DART model across a range of termination times (200-600 fs).

Our findings indicate that the pair-distance error remains consistently below 0.5 Å, which is significantly lower than the diffraction limit imposed by the experimental Q-range. This is further illustrated by comparing the pair-density difference under the same experimental constraints for a trajectory truncated at 350 fs versus the ground truth. The main features of the dissociation are well captured in this comparison.

The robustness of the DART model can be attributed to its ability to deterministically separate motions, where ballistic dissociation and diatomic rotation exhibit well-defined geometric characteristics, such as a straight line in 3D or a circle in 2D. Additionally, the scattering signal from dissociative pairs is dominated by Fe-X pairs, which are 3.5-5 times stronger than C-X and O-X pairs. Since the Fe atom is closely correlated with the center of mass of $Fe(CO)_4$, fitting $\mathbf{r_4}$ serves as an excellent approximation for capturing the main contribution to the dissociative motion. In addition, inaccuracies for shorter pair distances do not affect the primary objective of the extrapolation procedure, which is to track the subsequent pair density dissociation dynamics, including the modulation observed at distances greater than 7 Å, as detailed in the main text.

Suppl. Note 3.

Simulating scattering from trajectories

After trajectory extrapolation was performed, we used the independent atom model and the static approximation [4] to simulate the scattering difference signal for each trajectory, taking its initial time step $\tau = 0$ as the ground state structure to subtract the scattering from, or the laser-off scattering pattern, and then averaged among all trajectories:

$$\Delta S_0(Q,\tau) = \frac{1}{N}\sum_{k=1}^{N}\left(\sum_{b\neq a} f_a^*(Q) f_b(Q) j_0(QR_{ab}^{(k)}(\tau)) - \sum_{b\neq a} f_a^*(Q) f_b(Q) j_0(QR_{ab}^{(k)}(0)\right),$$

Where, N is the number of trajectories, and $R_{ab}^{(k)}(\tau)$ is the pair distance of the a-b atom pair at delay τ of the k-th trajectory. The comparison between the measured and simulated scattering is described in Suppl. Fig. 1.

To compare the anisotropy of the experimental scattering signal to the trajectory information requires knowledge of a dipole moment transition direction. Here, we tested with a case where such a transition will be along the axial direction, and along the equatorial direction for the case of an axial dissociation that is more prevalent in the simulation (90 out of 103 trajectories showed an axial dissociation direction). We calculated and averaged $\Delta S2$ for all trajectories, the comparison is seen in Suppl. Fig.. 2. We observe that the scattering anisotropy is consistent with the axial direction being dominant at early times, with a weak albeit non-negligible dipole moment along the axial direction.

Suppl. Note 4.

Real-space inversion of scattering signals

We employed the Natural Scattering Kernel (NSK) method, detailed in [7], for both experimental and simulated scattering difference signals. This approach enabled the real-space inversion of the isotropic scattering difference signal, $\Delta S_0(Q,\tau)$, to accurately recover the isotropic pair density. However, the fidelity of the anisotropic scattering difference $\Delta S_2(Q,\tau)$ was inadequate for extracting meaningful anisotropic pair density information.

Briefly, we resampled the scattering signal in Q bins with $\Delta Q = 0.1208\text{Å}^{-1}$ to improve signal fidelity, where the i-th bin is: $Q_i = \frac{j_{0i}}{j_{0N}} N\Delta Q$, where $Q_{max} = N\Delta Q = 4.34\text{Å}^{-1}$, sampled on j_{0i}, the i-th zero of the spherical Bessel function j_0. We applied a Lorch [8] window function $h(Q)$ to account for the finite detector range where the signal was detected $0.36<Q<4.34\text{Å}^{-1}$. We invert the isotropic scattering difference signal to obtain the pair density difference using:

$$\Delta PD_0(R,\tau)=\frac{[(M-1)\Delta q]^2}{j_{0M}}\sum_{i=1}^{M-1}\boldsymbol{G}_{mi}\frac{\Delta S_0(Q_i,\tau)h(Q_i)Q_i}{f_e(Q_i)},$$

Where $f_e(Q_i)=\sum_{b\neq a} f_a(Q_i)f_b(Q_i)$ is the effective form factor, and the transformation kernel is given by:

$$[\boldsymbol{G}]_{mi} = 2\frac{j_0\left(\frac{j_{0m}j_{0i}}{j_{0M}}\right)}{j_{0M}j_1^2(j_{0i})}, \tag{1}$$

where $j_1(j_{0i})$ is the first-order spherical Bessel function evaluated on the ith zero of j_0. The inverted ΔPD is distorted by the finite Q detection range and sample thickness. We from an NSKs dictionary to account for the measurement constraints, along the real space discretization given by $R_m=\frac{j_{0m}}{M\Delta Q}$, $m = 1\ldots M$, with M the number of grid points given by M=ceil($\pi/(\Delta R\Delta Q)$)+1, using ΔR=0.1Å:

$$NSK(R_m)=\frac{[(M-1)\Delta Q]^2}{j_{0M}}\sum_{i=1}^{M-1}\boldsymbol{G}_{mi}\tilde{j}_0(Q_iR_m)Q_i.$$

The distorted spherical Besssel function $\tilde{j}_0(Q,R_m)$ is given by :

$$\tilde{j}_0(Q,R_m)=\int_{z_0-dz}^{z_0+dz} dz\, h(Q(z))\, j_0(Q(z)R_m).$$

Where,

$$Q(z) = \frac{4\pi}{\sqrt{2}\lambda}\sqrt{1-\frac{z}{\sqrt{r_d^2+z^2}}},$$

expresses the relation between the scattering vector magnitude Q, the distance of the sample from the detector z, and the radial position on the detector $r_d = \sqrt{x^2 + y^2}$, and we used a sample thickness of 2 mm, and detector distance of z_0=74 mm.

The NSK dictionary $\boldsymbol{\mathcal{D}}$ is an array composed of NSKs at the real space sampling R_m:

$$\boldsymbol{\mathcal{D}} = \begin{bmatrix} | & & | & & | \\ NSK(R_1) & \cdots & NSK(Rm) & \cdots & NSK(R_M) \\ | & & | & & | \end{bmatrix}.$$

We use the NSKs dictionary to explain the inversion signal that is distorted by the measurement constraints, assuming the linear model:

$$\Delta PD_0 = \boldsymbol{\mathcal{D}}\mathbf{w},$$

Where we seek to estimate the weights vector $\mathbf{w}$ to recover the pair density difference described by $\Delta\rho(R) = \sum_m \mathrm{w}_m \delta(R - R_m)$. We do so using ℓ_2 regularization [9] solving ,

$$\min_{\mathrm{w}} \left\| \Delta PD_0 - \boldsymbol{\mathcal{D}}\mathbf{w} \right\|^{\mathbf{2}} + \epsilon \sum_{\mathbf{m}} |\mathrm{w}_{\mathbf{m}}|^{\mathbf{2}}$$

This has the analytic closed-form linear solution:

$$\mathbf{w} = (\boldsymbol{\mathcal{D}}^{\mathbf{T}}\boldsymbol{\mathcal{D}} + \epsilon\mathbf{1})^{-\mathbf{1}}\boldsymbol{\mathcal{D}}^{\mathbf{T}}\boldsymbol{\Delta}\mathbf{PD_0}$$

that can be solved using singular value decomposition. The regularization magnitude is given by ϵ, which can be estimated using the L-curve method or by cross-validation [10, 11].

Suppl. Note 5.

Kinetic model fit for first and second CO dissociation

The kinetic rate-model fit used for the fit in Fig 3a of the main paper is similar to the one used in [12] and was performed with the following set of equations:

$$\begin{aligned}
-\langle \Delta PD(R,\tau) \rangle_\alpha &= N_{(CO)1}(\tau) + N_{(CO)2}(\tau), \\
N_{(CO)1}(\tau) &= F_s(A, \tau_1, \tau_0, \tau), \\
N_{(CO)2}(\tau) &= F_s(A, \tau_1, \tau_0, \tau) - F_d(A, \tau_1, \tau_2, \tau_0, \tau), \\
F_s(A, \tau_1, \tau_0, \tau) &= A\left[1 + \mathrm{erf}\left(\frac{2\sqrt{\ln 2}}{\tau_1}(\tau - \tau_0)\right)\right], \\
F_d(A, \tau_1, \tau_2, \tau_0, \tau) &= \exp^{-(\tau - \tau_0 - \kappa)/\tau_2} F_s(A, \tau_1, \tau_0, \tau - 2\kappa).
\end{aligned}$$

where $-\langle \Delta PD(R,\tau) \rangle_\alpha$ is the average pair density measured around the Fe-C steady stage bond distance (region α : $1.3 < R < 2.3$Å) where the sign is reversed to account for CO production, $N_{(CO)i}$ are the fitted dissociation products of the i-th CO. The first dissociation, or the production of $N_{(CO)1}(\tau)$ is modeled in terms of cumulative growth, or the time integral of a Gaussian, resulting in the error function (erf), a dissociation onset time delay τ_0, and the time constants τ_1. The second dissociation model the exponential decay of $Fe(CO)_4$, or the production of $N_{(CO)2}$, with an amplitude factor A, a τ_2 time constant, and with $\kappa = \frac{\tau_1^2}{16\tau_2 \ln 2}$.

Suppl. Note 6.

Estimation of the dissociation velocity and rotation frequency

6.1 From simulation:

The dissociation velocity for each trajectory is determined by calculating the average velocity post-dissociation between the center of mass of the dissociating CO and that of the remaining $Fe(CO)_4$. Additionally, the rotation frequency of CO, ω_{CO}, is derived from the DART model, as detailed in Section 2. In Suppl. Fig. 7 we show how the probability histograms are generated and analyzed for both the dissociation velocity and rotation frequency. Each histogram is fitted with a generalized extreme value distribution probability function, chosen for its effectiveness in capturing the shape of the data distributions.

To delve deeper into the interplay between these variables, a 2D histogram is constructed, which not only reveals a correlation between dissociation velocity and rotation frequency but also captures the energy distribution between translational and rotational motion. This analysis indicates that the energy is partitioned between these motions during the dissociation process. These results are described in Suppl. Fig. 7c.

6.2 From experimental ΔPD:

We initially estimate the dissociation velocity as the slope calculated between the onset of dissociation for the Fe-C pair and the point at which it manifests as density modulation, occurring around 400-600 fs and at distances of 7-8 Å. The onset time of dissociation is determined using a kinetic model fit within the 25-150 fs time delay window, based on a steady-state Fe-C pair distance of 1.8 Å. The density modulation in the measured ΔPD is identified through a smoothing procedure and by applying a threshold to the experimental data. All potential slopes between the dissociation onset and the observed density modulation are weighted by the measured ΔPD intensity at the modulation, and fitted with a generalized extreme value probability distribution, which effectively captures the histogram, as depicted in Suppl. Fig. 8. The average values and uncertainties of these distributions serve as the estimated parameters reported.

The same methodology is applied to estimate the dissociation velocity following the second CO loss. In this instance, the dissociation time constant, as determined by the kinetic model, is longer (3 ps), resulting in a more diffuse temporal distribution of PD contributions at distances beyond 6.5 Å. Consequently, we interpret the PD accumulation within the range $6.5<R<8$ Å as arising from the Fe-C and Fe-O pairs following the second CO loss, which crosses the largest intramolecular O-O distances, leading to a "boiling-off" where their PD is statistical rather than ballistic. To estimate the dissociation velocity, we use the same steady-state Fe-C distance (1.8 Å) but account for a time shift after the completion of the first CO loss (150-250 fs). The reference point for PD accumulation is similarly identified within a corresponding time window at distances of $6.5<R<8$ Å. The results of this analysis, presented in Suppl. Fig. 9, should be regarded as an upper bound, given that the full distribution of dissociation times is not captured within the observed delay range.

6.3 Energy partition analysis of a dissociating and rotating CO Molecule

The translational Kinetic Energy (KE) given the dissociation velocity of the first CO loss $v = 11.2$Å/ps and the mass of a CO molecule, is calculated as:

$$KE_{CO1} = \frac{1}{2}mv^2 \approx 0.182\,\mathrm{eV}$$

The rotational Kinetic Energy (Er) is given by:

$$E_r = \frac{1}{2} I\omega^2 \approx 0.0175\,\mathrm{eV}$$

Where $I = \mu r^2$ is the moment of inertia, with the CO bond distance r and reduced mass μ. Similarly, the translational Kinetic Energy of the second CO loss given the estimated dissociation velocity $v = 8.6\text{Å}/ps$ is:

$$KE_{CO2} \approx 0.107\,\mathrm{eV}$$

Suppl. Note 7.

Normal mode analysis for $Fe(CO)_4$

Geometry optimization and normal mode computations for $Fe(CO)_4$ was carried out at the TPSSh/def2-TZVP level of theory [13, 14] using ORCA quantum chemical package [15] . The optimized geometries were confirmed to be stationary points by the absence of imaginary modes. The structures were optimized at three different electronic states, i.e. the triplet state, the closed shell singlet, and the open shell singlet followed by the corresponding normal mode analysis. The open shell singlet was optimized using the broken-symmetry approach. The ⟨S2⟩ value of the broken symmetry singlet state at the optimized geometry was found to be 1.005, which is consistent with an open-shell singlet state. Tables with the different modes and their parameters are detailed below:

Suppl. Table 1. Closed Shell Singlet (CS) normal modes.

Mode	freq (cm^{-1})	ϵ (L/(mol cm))	I (km/mol)	T^2 (a.u.)	T_X	T_Y	T_Z
6	42.02	0.000083	0.42	0.000615	-0.000011	0.000000	-0.024804
7	76.11	0.000203	1.02	0.000831	0.028835	-0.000000	-0.000130
8	79.48	0.000533	2.69	0.002094	-0.000088	0.000000	-0.045756
9	94.03	0.000001	0.01	0.000003	0.000000	0.001848	-0.000000
10	95.69	0.000391	1.98	0.001275	0.000000	0.035704	-0.000000
11	354.56	0.000174	0.88	0.000154	0.000000	0.012392	-0.000000
12	368.09	0.000460	2.33	0.000390	0.019750	0.000000	-0.000016

Suppl. Table 2. Open Shell Singlet (OS) normal modes.

Mode	freq (cm^{-1})	ϵ (L/(mol cm))	I (km/mol)	T^2 (a.u.)	T_X	T_Y	T_Z
6	54.55	0.000034	0.17	0.000195	0.000133	-0.000000	0.013970
7	77.75	0.000000	0.00	0.000000	0.000000	-0.000056	0.000000
8	81.07	0.000084	0.42	0.000322	0.017931	-0.000000	0.000101
9	85.78	0.000283	1.43	0.001030	0.000000	-0.032097	0.000000
10	91.14	0.000091	0.46	0.000313	-0.000041	0.000000	0.017689
11	339.08	0.000243	1.23	0.000224	0.000227	-0.000000	0.014962
12	342.23	0.000023	0.11	0.000021	0.004520	-0.000000	-0.000452

Suppl. Table 3. Triplet (Tr) normal modes.

Mode	freq (cm^{-1})	ϵ (L/(mol cm))	I (km/mol)	T^2 (a.u.)	T_X	T_Y	T_Z
6	61.74	0.000000	0.00	0.000002	0.000002	-0.000000	-0.001432
7	75.45	0.000000	0.00	0.000000	-0.000000	0.000043	0.000000
8	79.22	0.000108	0.55	0.000426	-0.020628	0.000000	-0.000044
9	83.93	0.000341	1.72	0.001268	-0.000000	-0.035610	0.000000
10	90.53	0.000097	0.49	0.000336	0.000010	-0.000000	-0.018332
11	330.19	0.000033	0.17	0.000032	-0.005623	-0.000000	-0.000010
12	332.35	0.000000	0.00	0.000000	-0.000000	0.000155	-0.000000

where ϵ is the Molar absorptivity, I is the integrated intensity, T^2 is the square of the transition dipole moment in atomic units, and T_X, T_Y, T_Z are Cartesian components of the transition dipole moment.

Note that only modes 6-10 are relevant to the primary frequency structure observed in the main paper (Fig 3d), as the modes prior to mode 6 are translational and rotational degrees of freedom, and do not affect pair density distances analyzed. Therefore, only modes 6-10 will be further analyzed. Suppl. Fig. 10 displays a description of the structural displacements for these modes across various configurations.

Relative energies of the optimized closed-shell singlet, open-shell singlet, and triplet states of $Fe(CO)_4$:

- Open-Shell (broken symmetry) singlet: -1717.321650178036 (7.1 kcal/mol)
- Closed-shell singlet: -1717.322479714234 (7.6 kcal/mol)
- Triplet: -1717.333805474550 (lowest)

7.1 Determining the Contribution of Modes to Experimental Observations

The structural dynamics of modes 6 through 10 were analyzed to trace the variations in pair-distance motions. It was observed that atom pairs of the same type, such as the four Fe-C pairs, display distinct pair distance dynamics depending on the mode influencing them. For instance, by examining the motion of the Fe-C pairs across each mode shown in Suppl. Fig. 11, it becomes apparent that certain modes, like the 9th mode in the closed shell singlet configuration, involve relatively minor movements. In contrast, other modes demonstrate a broader range of motion. Additionally, when pairs of the same type (Fe-C) move in opposite directions, there is a tendency to increase the amplitude of higher frequencies within specific distance ranges. For example, the 8th mode of the closed shell (CS) singlet configuration, compared to the singlet open shell (OS) and triplet (Tr) configurations. For the OS and Tr configurations, we observe movements in opposite directions of pairs, that effectively double the frequency of the total pair distance motions within the 1.82 Å range. As a result, the overall Fe-C pair density in this range can exhibit motions that may have a frequency of up to 192 cm^{-1}, even though the maximal eigen-frequency of modes 6-10 is 96 cm^{-1}, as also seen in Suppl. Fig. 12 detailed below.

For each mode i=6...10 in each configuration j=$\{CS, OS, Tr\}$ we simulated the scattering signal and the inverted pair density under the same experimental conditions $PD_k(R,\tau)$, where $k = \{i,j\}$ encodes the mode and configuration. We averaged these in the same experimental pair distances range (α : 1.3<R<2.3Å) and Fourier transformed to obtain $\langle PD_k(f)\rangle_\alpha$. We conducted a best-fit analysis for the case of each configuration and a case of a mixture of all configurations. The results for each case are described in Suppl. Fig. 12.

We discover that the frequency peaks at 31 and 78 cm^{-1} correspond most closely with the 6th and 8th normal modes of the CS configuration. In contrast, the frequency range from 100 to 200 cm^{-1} is best explained by both the OS and Tr configurations. Employing a mixture model that allows contributions from all configurations provides the most accurate fit. This model partitions the contributions as follows: 57% CS, 29% OS, and 14% Tr. The best-fit procedure was done by minimizing the regularized least squares:

$$\begin{aligned} \text{minimize} \quad & \|Ax - \delta(f)\|^2 + \epsilon \sum_{k=1}^{n} x_k \\ \text{subject to} \quad & x_k \geq 0, \quad k = 1, \ldots, n \end{aligned}$$

using the method described in [16], where A is an array of all $\langle PD_k(f) \rangle_\alpha$ included in the fit, x are non-negative weights capturing contributions of different modes and configurations, ϵ is the regularization parameter that is found using cross-validation, and $\delta(f)$ is Fourier transform of $\delta(\tau)$, the residual between the averaged measured PD in range α and the fitted kinetic model from Section 5, given by:

$$\delta(\tau) = -\langle \Delta PD(R, \tau) \rangle_\alpha - (N_{(CO)1}(\tau) + N_{(CO)2}(\tau))$$

In addition, we have used for the fitting procedure the frequency range $0 < f < 200$ cm^{-1} as explained in the previous paragraph regarding the doubling of the effective frequency on this range.

Suppl. Note 8.

Estimation of the experimental instrument response function

The Instrument Response Function (IRF) characterizes the temporal resolution of the measurement, detailing how the experimental apparatus system responds to a faster input signal. In the context of time-resolved measurements scattering, the IRF essentially describes the "blurring" effect of the instrument on the theoretically instantaneous events being measured, such as the atomic pair distance oscillations following photo-excitation. We estimate the IRF and the excitation fraction by fitting the average of all simulated trajectories ΔPD to the experimental ΔPD around the β region of pair distances, where the oscillations are best observed (Suppl. Fig. 13). The parameters of the fit are the time zero offset, an overall scaling factor, and the FWHM of a Gaussian response function that was convolved with the simulated pair density. We obtain from the fit a Gaussian response function with a FWHM of 58 ± 7 fs.

Suppl. Note 9.

Laser pulse energy scan

We obtained scattering difference patterns as a function of the UV laser pulse fluence in the range 0.67-3.6 μJ, integrated across the Q range $2.4 < Q < 4\text{Å}^{-1}$, that contain only positive scattering difference signals. Suppl. Fig. 14 shows that we obtained a linear response for the two lowest pump pulse energies, and a saturation response at pulse energies $> 2\mu$J. For the pump-probe experiment, we have used the lowest energy 0.67μJ. To validate we are in the linear regime, we further estimated the optical density and the excitation density.

Starting with the experimental conditions, a vapor pressure of 3 Torr, excitation wavelength of 266 nm, 58 fs pulses, focused to an estimated diameter of 60 μm, which was achieved by focusing the laser beam toward the downstream end of the scattering cell, as detailed in a similar study [17].

The single-photon optical cross section of $Fe(CO)_5$ at 266 nm is $\sigma_1 \approx 6.75{\times}10^{-18}\,\text{cm}^2$ [18]. The laser cross-sectional area is $A = \pi r^2 \approx 2.83 \times 10^{-5}\,\text{cm}^2$, the effective path length is L≈2 mm, and the interaction volume $V = A \times L \approx 5.65 \times 10^{-6}\,\text{cm}^3$. To estimate the number density of $Fe(CO)_5$ at 3 Torr we use the ideal gas law, $n{=}\frac{p}{k_B\,T} \approx 9.73 \times 10^{16}\,\text{cm}^{-3}$, with the number density n at pressure p and temperature T.

Using the Beer-Lambert optical depth over path length: OD$=n\,\sigma_1\,L{\approx}0.131$. Hence, the fraction of photons absorbed in a linear Beer-Lambert sense is estimated by $1{-}e^{-0.131}{=}12.3\%$, a value in the 10% range, supporting the linear regime assumption. The excitation density can be estimated by $N_{ph} \times (1{-}e^{-0.131})/V \approx 1.95 \times 10^{16}\,\text{cm}^{-3}$ where $N_{ph} \approx 9 \times 10^{16}$ is the number of photons per pulse, which is $\approx$ 20% the sample density, well below saturation that is expected for values approaching 100%. In addition, we made the assumption that the x-ray and laser beams are perfectly overlapped, with a uniform radial intensity profile for the laser beam, while in practice, the measured excitation fraction is expected to be smaller in light to these assumptions.

Suppl. Note 10.

Supplementary Figures

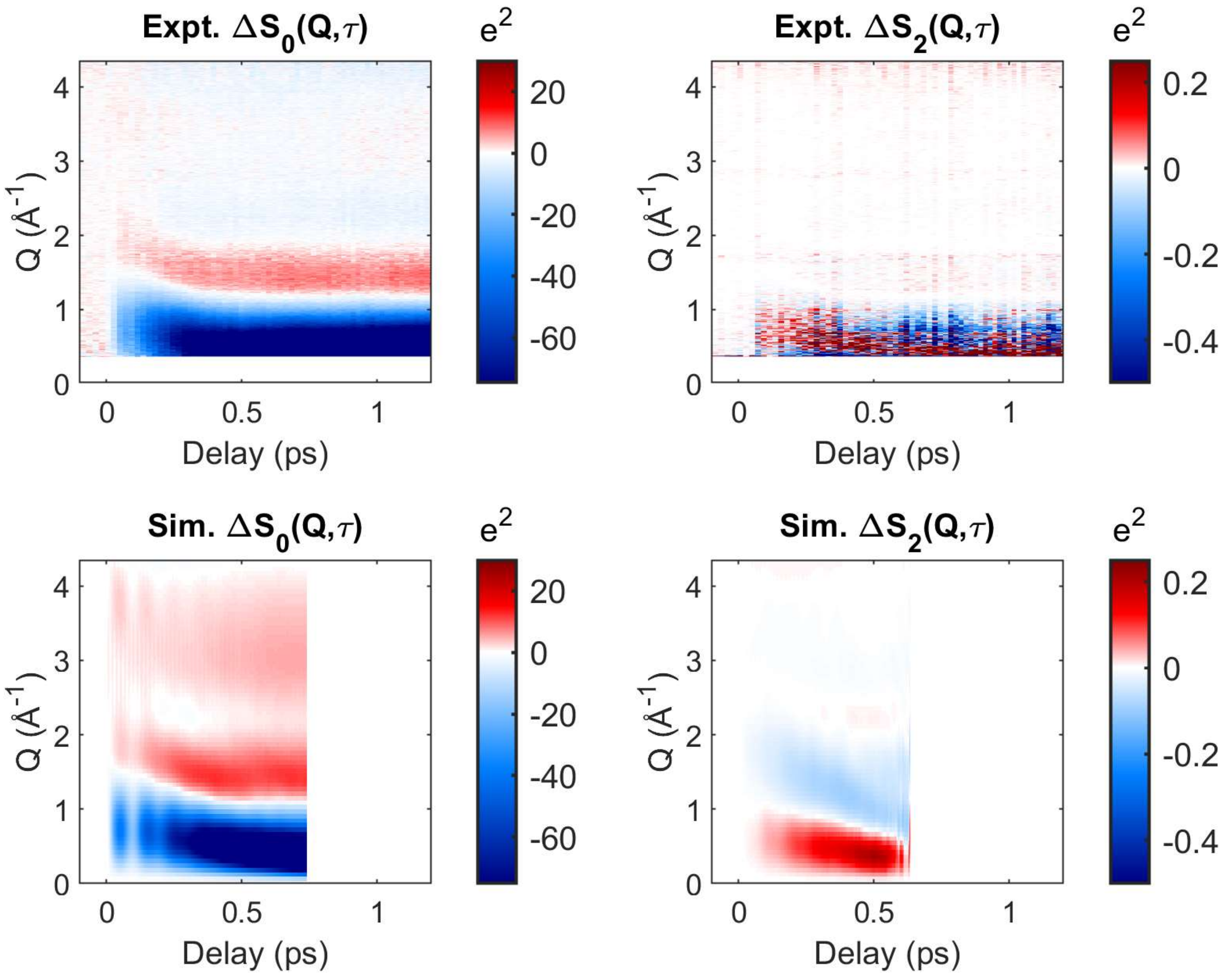

Suppl. Fig. 1. Experimental and simulated scattering difference curves for the isotropic scattering component ΔS_0, and the anisotropic scattering component ΔS_2 in (e^2) units. The simulated scattering is based on the averaging of all trajectories, but without considering the instrument response function. For the anisotropic scattering case, the simulated scattering was scaled to the experimental one, and the limited signal fidelity allows comparison only for the lower Q range.

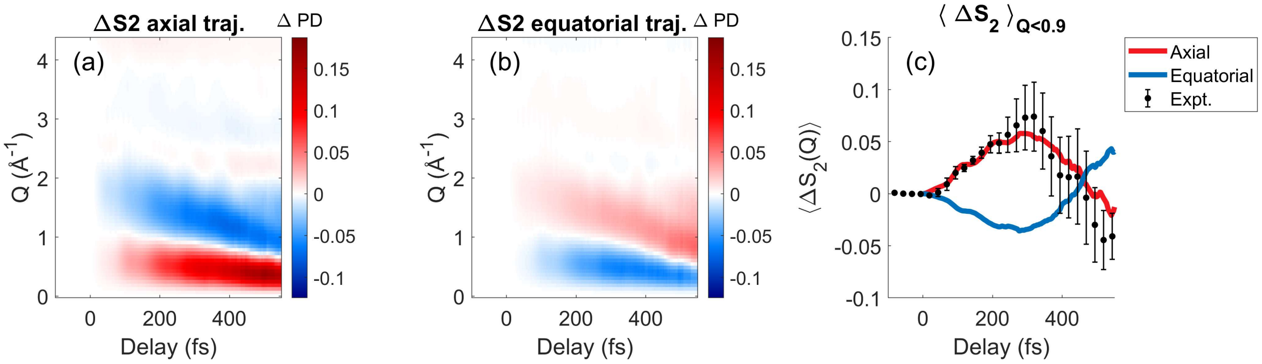

Suppl. Fig. 2. The simulated anisotropic scattering difference for the case of (a) an axial dipole direction versus (b) an equatorial dipole direction. (c) The scattering is averaged for the range $Q < 0.9\text{Å}^{-1}$ (error bars represent the standard deviation of this average), where it can be compared with the experimental scattering anisotropy signal. This range also encodes large distances motion, consistent with the elongation of the Fe-C bond and dissociation. We obtain that the axial direction is consistent with the early time dynamics of the experimental signal.

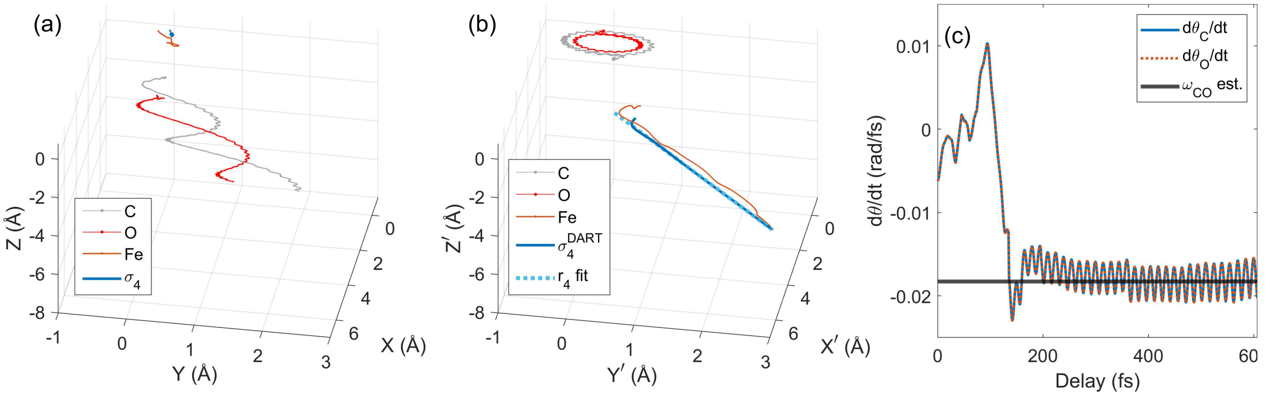

Suppl. Fig. 3. The DART model used for extrapolating the dissociative pairs. (a) The XYZ positions of the dissociating CO, the Fe atom, and σ_4 the center of mass of $Fe(CO)_4$ are plotted for a single trajectory. The traces of the CO trajectory exhibit rotation while dissociation takes place, while σ_4 exhibits negligible motion. (b) Transforming the trajectory information to a new frame of reference X′Y′Z′ co-moving with the center of mass of the dissociating CO is done as part of the DART model. This allows us to fit the dominant dissociation dynamics governed by the position and direction of σ_4, as evidenced by the Fe position in this frame. (c) The DART frame facilitates obtaining the CO rotation frequency ω_{CO}, estimated by taking the time derivative of the azimuthal angle of the rotating CO at the range $\tau > \tau_d$. The fast oscillatory signal is due to CO vibration that is also present.

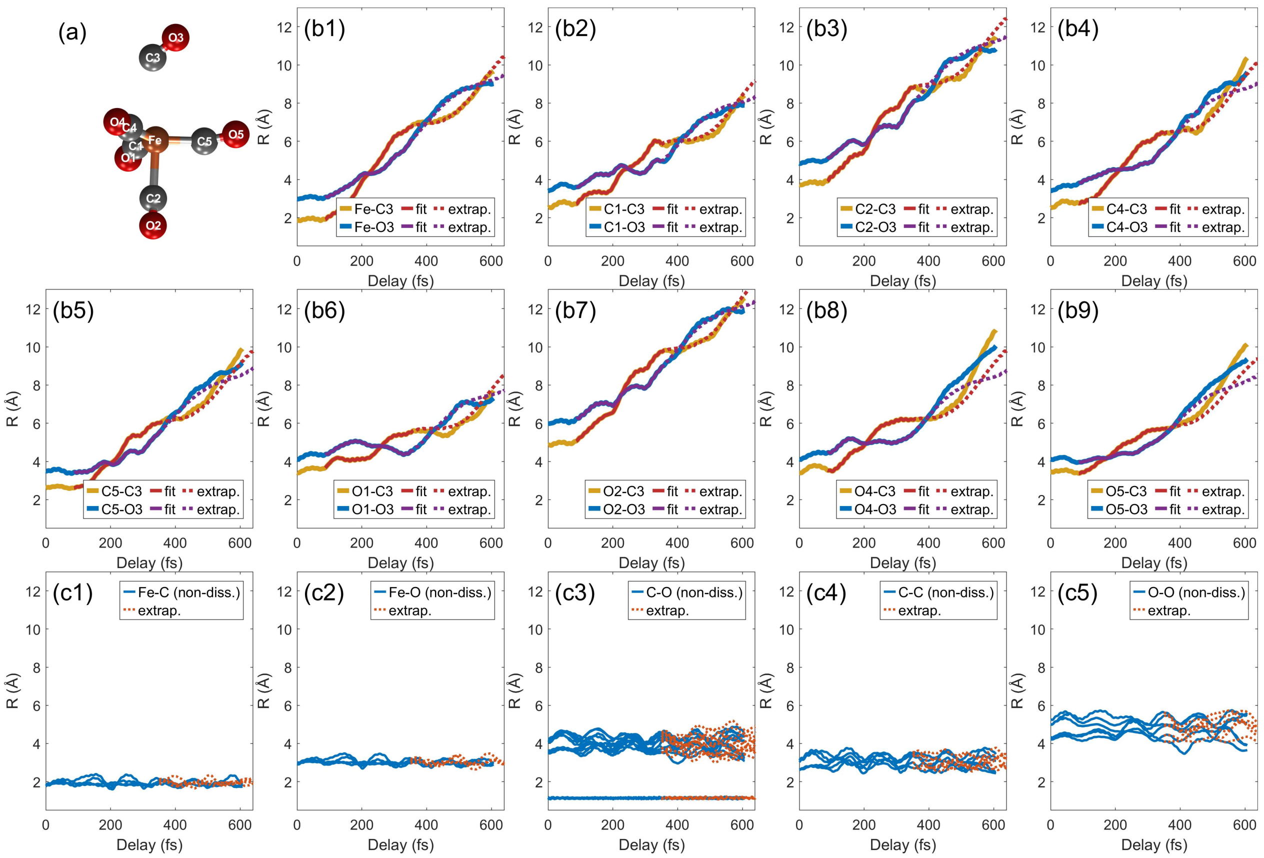

Suppl. Fig. 4. Extrapolating atomic pair distances for trajectories. (a) A snapshot of $Fe(CO)_5$ structure with atom labels, showing dissociation in the axial direction. (b) The dissociative pairs are extrapolated by applying the DART model (see text). The fit procedure only uses the first 350 fs of the trajectory and the fit parameters are used to extrapolate to 640 fs (dashed lines). The extrapolated pair distances are compared to the computed trajectory distances that had a termination time of 608 fs, validating the model's ability to capture the CO dissociation and rotational motions, which are evident as alternating crossings and separations of the dissociative pairs. (c) The non-dissociative pairs, organized by type, are also extrapolated based on the first 350 fs, using Burg's method (see text).

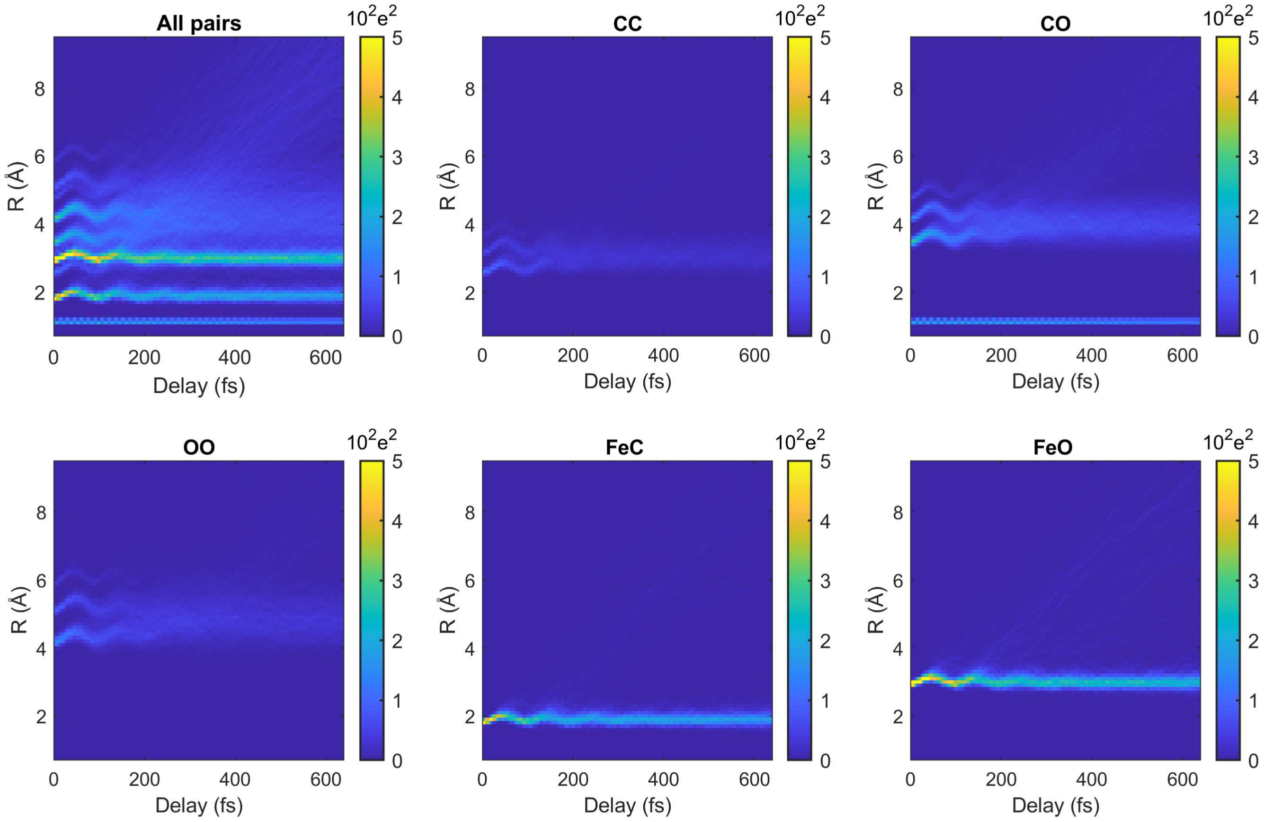

Suppl. Fig. 5. The time-dependent pair density, averaged across all trajectories, is calculated following extrapolation up to the duration of the longest-lived trajectory (640 fs). This calculation is accompanied by a detailed analysis of the various types of atomic pairs. The color bar represents pair density. While motion initiates from changes in Fe-C bond distances, it influences other atomic pairs that act as ”spectators” as explained by the spectator effect concept.

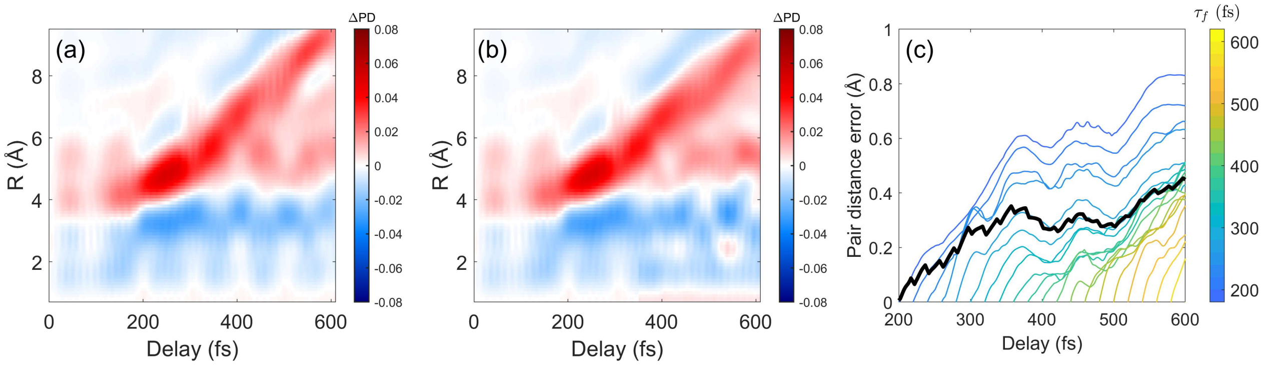

Suppl. Fig. 6. We compared (a) the calculated pair density difference for a trajectory with a termination time of 608 fs, with (b) a scenario where all pairs of that trajectory were extrapolated after a 350 fs delay, as detailed in the text. The comparison shows good agreement in describing the dissociation details, particularly in capturing the observed density modulations associated with rotational motion at distances >7Å. (c) For the dissociative pairs, the accumulated weighted error as a function of the trajectory termination time, denoted by τ_f, is calculated for increasing termination times between 200-600 fs. We obtain that the average accumulated error is less than 0.5 Å, well below the diffraction limit imposed by the experimental Q-range. In this analysis, trajectory termination times are assumed to be uniformly distributed, consistent with the distribution of the trajectories used.

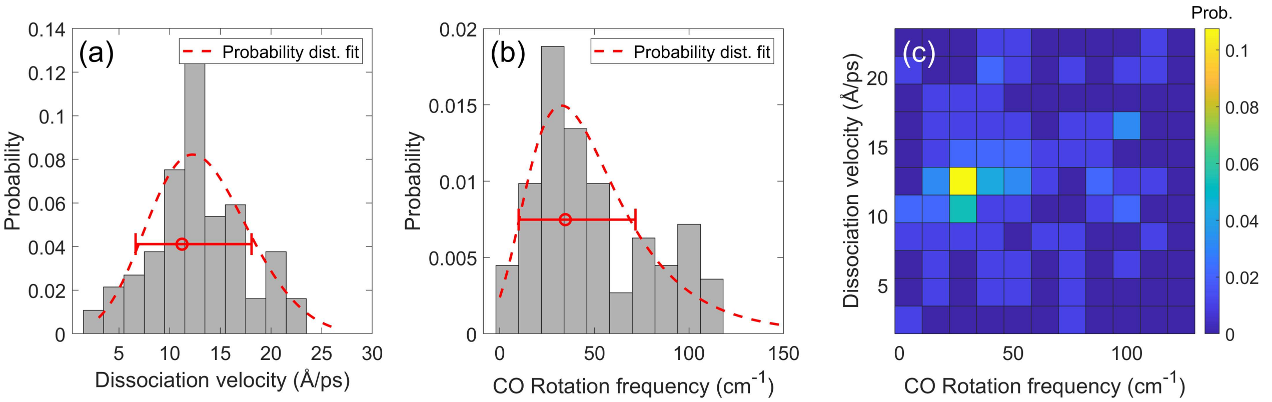

Suppl. Fig. 7. (a) A histogram of the dissociation velocity obtained from all trajectories, calculated as the average velocity after dissociation between the center of mass of the dissociating CO and the center of mass of the remaining $Fe(CO)_4$. The histogram is fitted with a generalized extreme value distribution probability function, where the average and uncertainties of the fit are used as the estimated dissociation velocity (red error bar). (b) The same is done for the CO rotation frequency, obtained from the DART model fit. (c) a 2D histogram showing the correlation between translation and rotation during the dissociation.

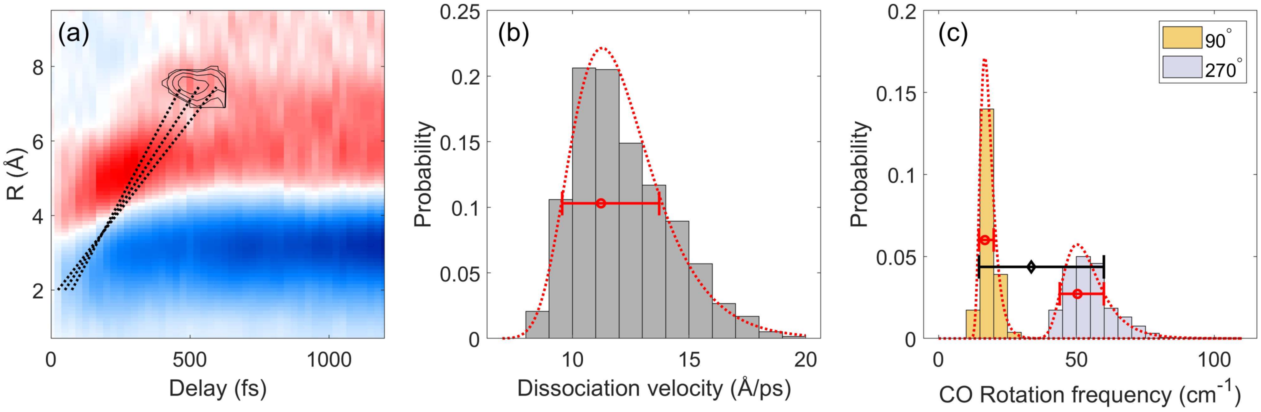

Suppl. Fig. 8. (a) The dissociation velocity and rotation frequency are obtained by weighting all the possible slopes (dashed lines) from the onset of dissociation given by the kinetic model at the Fe-C steady state position, and the density modulation (contour lines) that is found by smoothing and applying a threshold to the experimental ΔPD. (b) A histogram of the dissociation velocities is obtained from the found slopes. We fitted the histogram with a generalized extreme value distribution probability function, where the average and uncertainties of the fit are used as the estimated dissociation velocity (red error bar). (c) The same is done for the rotation frequency assuming a 90° or 270° rotation that can explain the density modulation. Since both possibilities are valid, we averaged their mean probability distribution as the estimated rotation frequency, and used their uncertainty bounds, as the bounds for the total uncertainty in the analysis (black error bar).

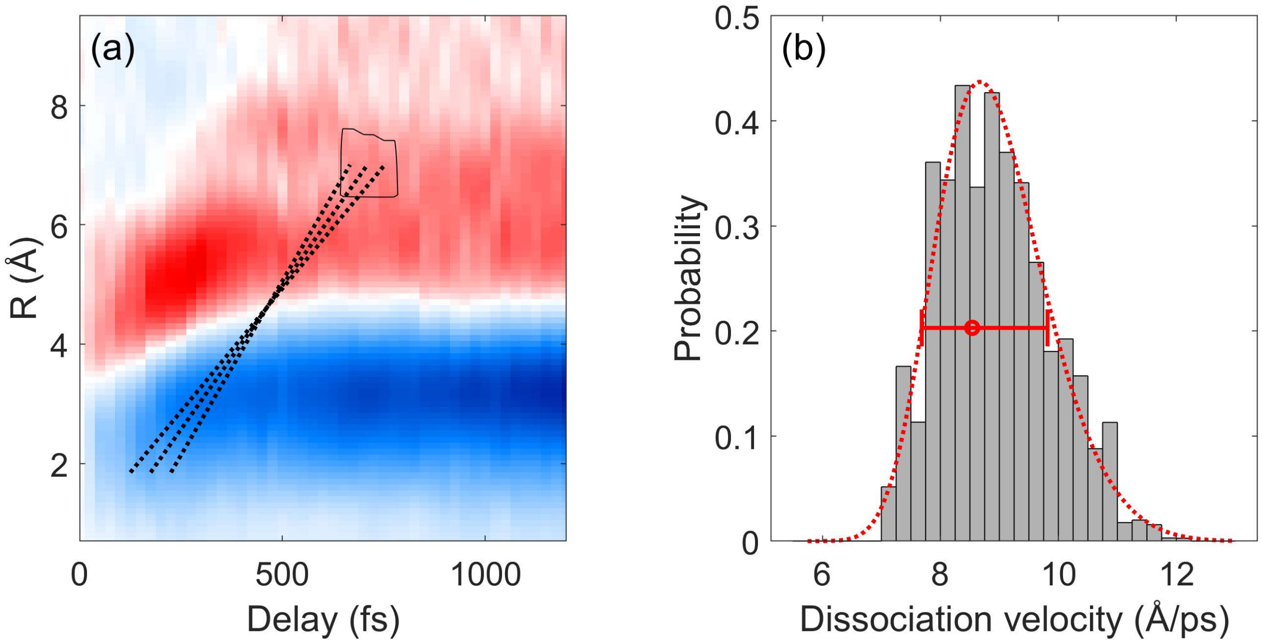

Suppl. Fig. 9. (a) The dissociation velocity of the second CO is estimated by weighting the possible slopes (dashed lines) from a delay window that begins after the first CO loss that was found by the kinetic model (150-250 fs) at the Fe-C steady state position, and the density build-up (contour lines) at pair distances that are larger than the $Fe(CO)_4$ intramolecular distances, that is found by smoothing and applying a threshold to the experimental ΔPD. (b) A histogram of the dissociation velocities is obtained from the found slopes. We fitted the histogram with a generalized extreme value distribution probability function, where the average and uncertainties of the fit are used as the estimated dissociation velocity (error bar).

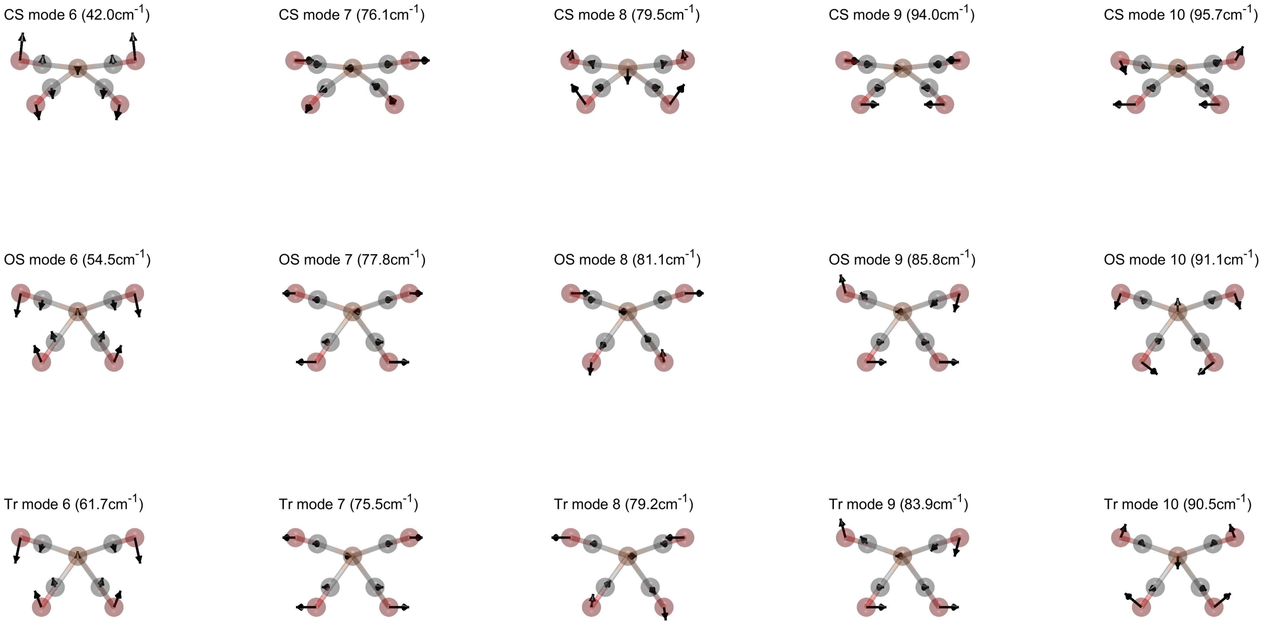

Suppl. Fig. 10. Structures and displacements (arrows) of the various normal modes and configurations of $Fe(CO)_4$.

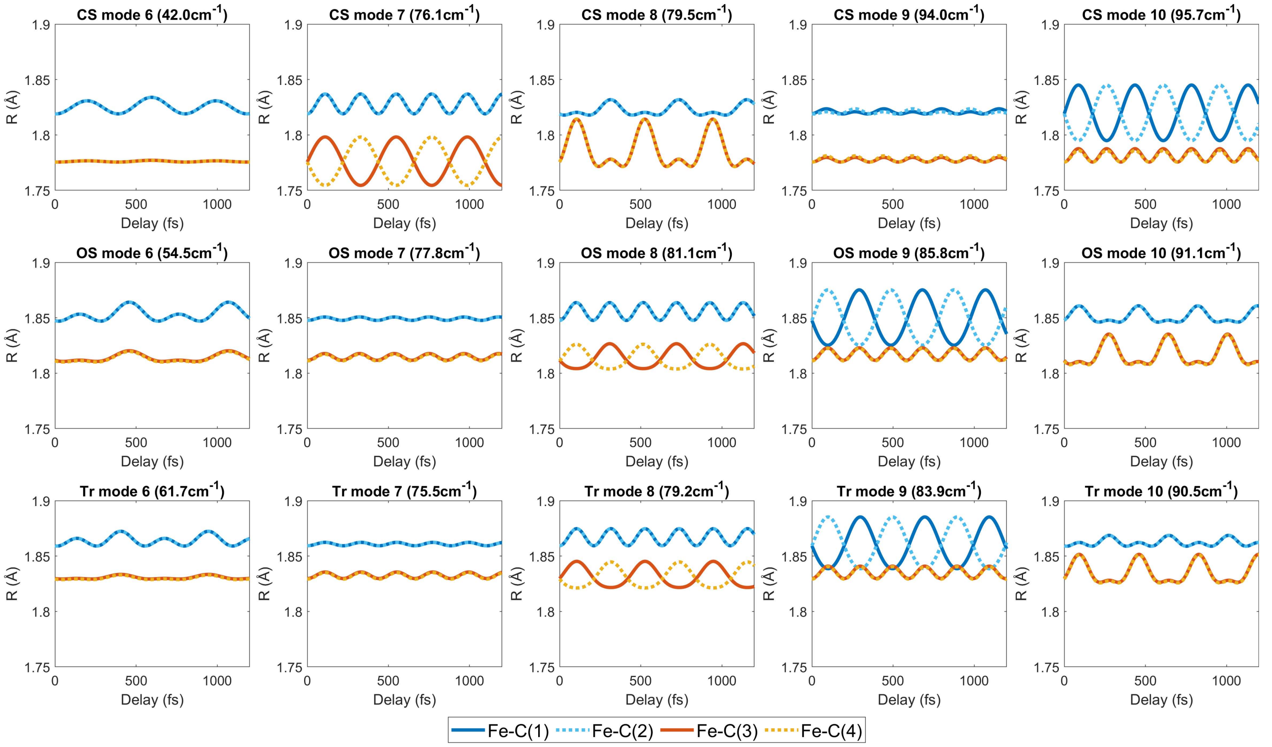

Suppl. Fig. 11. Fe-C pair distance motion for the 6-10 normal modes of singlet closed shell (CS), singlet open shell (OS), and triplet (Tr) configurations. Note that though each normal mode corresponds to a distinct eigen-frequency, different pairs that move in opposite directions may produce higher frequencies in a given distance range. For example, for the 6th mode of the CS configuration, the frequency of all Fe-C pairs is predominantly the eigen-frequency, while for the CS 7th mode, Fe-C3 and Fe-C4 pairs move in an opposite direction relative to each other producing a doubling of the frequency in the distance range.

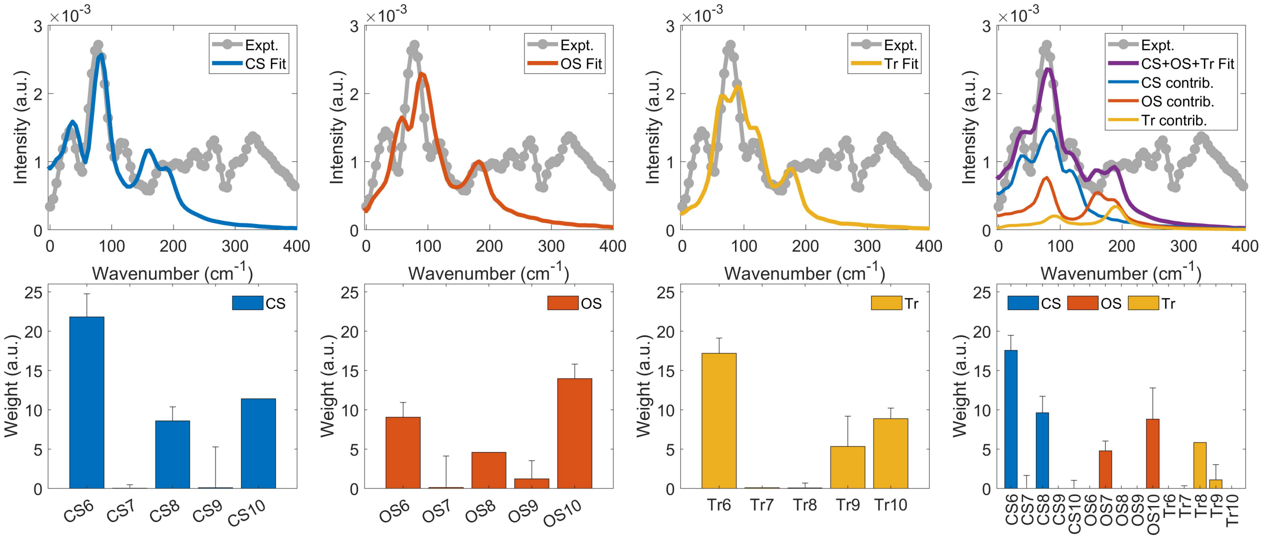

Suppl. Fig. 12. Finding the normal mode contribution of the singlet closed shell (CS), singlet open shell (OS), and triplet (Tr) to the overall measured spectrum that was obtained in Fig 3d of the main paper capturing Fe-C dynamics of the remaining $Fe(CO)_4$. Fitting with each configuration reveals that the CS 6th and 8th normal modes best capture the experimental peaks at 31 and 78 cm^{-1}, while both OS and Tr can explain the peaks at higher frequencies. Using a mixture model where all configurations may contribute achieves the best fit, where their relative contributions are 57% CS, 29% OS, 14% Tr. The uncertainties (error bars) of the different weights were estimated via bootstrap resampling, which involves repeatedly sampling with replacement from the original dataset, creating 100 bootstrap datasets, recalculating the weights of each normal mode for these samples, and deriving error bars from the resulting distribution to quantify the uncertainty in the original estimates.

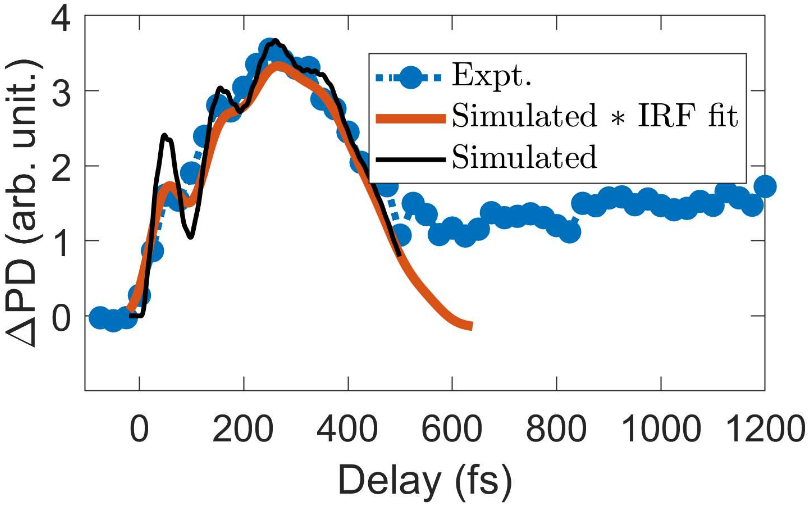

Suppl. Fig. 13. Estimation of the experimental instrument response function. The measured averaged ΔPD at range β ($3.7<R<7$Å) versus the simulated ΔPD with and without the IRF fit.

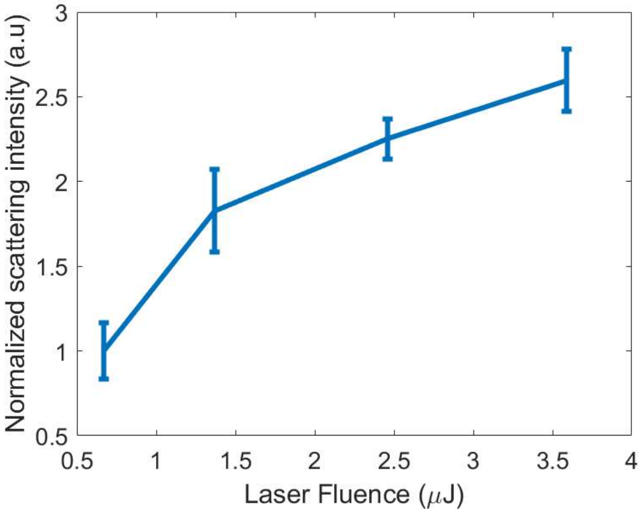

Suppl. Fig. 14. UV pump pulse energy scan as a function of scattering intensity on the detector in the range $2.4 < Q < 4\text{Å}^{-1}$. The error bars represent the standard deviation obtained from an analysis of detector signal variance for each UV pump energy in the Q range used.

Supplementary References

[1] Adi Natan et al. "Resolving multiphoton processes with high-order anisotropy ultrafast X-ray scattering". In: *Faraday Discussions* 228 (2021), pp. 123–138.

[2] P Bösecke and O Diat. "Small-angle X-ray scattering at the ESRF high-brilliance beamline". In: *Journal of Applied Crystallography* 30.5 (1997), pp. 867–871.

[3] Greg Hura et al. "A high-quality x-ray scattering experiment on liquid water at ambient conditions". In: *The Journal of Chemical Physics* 113.20 (2000), pp. 9140–9148.

[4] Ulf Lorenz, Klaus B. Møller, and Niels E. Henriksen. "Theory of time-resolved inelastic x-ray diffraction". In: *Phys. Rev. A* 81.2 (Feb. 2010), p. 023422. DOI: 10.1103/PhysRevA.81.023422. URL: http://link.aps.org/doi/10.1103/PhysRevA.81.023422 (visited on 08/03/2016).

[5] Jeremy J. Gray. "Olinde Rodrigues' Paper of 1840 on Transformation Groups". In: *Archive for History of Exact Sciences* 21.4 (1980), pp. 375–385. ISSN: 00039519, 14320657. URL: http://www.jstor.org/stable/41133613 (visited on 08/19/2024).

[6] Steven M Kay. *Modern spectral estimation*. Pearson Education India, 1988.

[7] Adi Natan. "Real-space inversion and super-resolution of ultrafast scattering". In: *Phys. Rev. A* 107 (2 Feb. 2023), p. 023105. DOI: 10.1103/PhysRevA.107.023105. URL: https://link.aps.org/doi/10.1103/PhysRevA.107.023105.

[8] E Lorch. "Neutron diffraction by germania, silica and radiation-damaged silica glasses". In: *Journal of Physics C: Solid State Physics* 2.2 (1969), p. 229.

[9] Gene H Golub, Per Christian Hansen, and Dianne P O'Leary. "Tikhonov regularization and total least squares". In: *SIAM journal on matrix analysis and applications* 21.1 (1999), pp. 185–194.

[10] Per Christian Hansen and Dianne Prost O'Leary. "The use of the L-curve in the regularization of discrete ill-posed problems". In: *SIAM journal on scientific computing* 14.6 (1993), pp. 1487–1503.

[11] Sylvain Arlot and Alain Celisse. "A survey of cross-validation procedures for model selection". In: *Statistics surveys* 4 (2010), pp. 40–79.

[12] Ph. Wernet et al. "Communication: Direct evidence for sequential dissociation of gas-phase $Fe(CO)_5$ via a singlet pathway upon excitation at 266 nm". In: *The Journal of Chemical Physics* 146.21 (June 2017), p. 211103. ISSN: 0021-9606. DOI: 10.1063/1.4984774. eprint: https://pubs.aip.org/aip/jcp/article-pdf/doi/10.1063/1.4984774/13595045/211103_1_online.pdf. URL: https://doi.org/10.1063/1.4984774.

[13] Jianmin Tao et al. "Climbing the Density Functional Ladder: Nonempirical Meta–Generalized Gradient Approximation Designed for Molecules and Solids". In: *Phys. Rev. Lett.* 91 (14 Sept. 2003), p. 146401. DOI: 10.1103/PhysRevLett.91.146401. URL: https://link.aps.org/doi/10.1103/PhysRevLett.91.146401.

[14] Florian Weigend and Reinhart Ahlrichs. "Balanced basis sets of split valence, triple zeta valence and quadruple zeta valence quality for H to Rn: Design and assessment of accuracy". In: *Phys. Chem. Chem. Phys.* 7 (18 2005), pp. 3297–3305. DOI: 10.1039/B508541A. URL: http://dx.doi.org/10.1039/B508541A.

[15] Frank Neese et al. “The ORCA quantum chemistry program package”. In: *The Journal of Chemical Physics* 152.22 (June 2020), p. 224108. ISSN: 0021-9606. DOI: 10.1063/5.0004608.

[16] Seung-Jean Kim et al. “An interior-point method for large-scale ℓ_1-regularized least squares”. In: *IEEE journal of selected topics in signal processing* 1.4 (2007), pp. 606–617.

[17] Brian Stankus et al. “Advances in ultrafast gas-phase x-ray scattering”. In: *Journal of Physics B: Atomic, Molecular and Optical Physics* 53.23 (2020), p. 234004.

[18] T. Aylett et al. “Optical properties of meteoric smoke analogues”. In: *Atmospheric Chemistry and Physics* 19.19 (2019), pp. 12767–12777. DOI: 10.5194/acp-19-12767-2019. URL: https://acp.copernicus.org/articles/19/12767/2019/.
