# Supplementary material for: Real-space observation of the dissociation of a transition metal complex and its concurrent energy redistribution
Source: Nat Commun. 2025 May 22;16:4767. doi: 10.1038/s41467-025-60009-8 (PMC12098838; doi:10.1038/s41467-025-60009-8)
Supplement: Supplementary file 2 — Description of Additional Supplementary Files [file 41467_2025_60009_MOESM2_ESM.pdf]

Description of Additional Supplementary Files:

Supplementary Data 1: This dataset includes experimental and simulated data, along with codes, all related to the study. The data can be explored by running main.m. Detailed descriptions of the file contents and structure are provided in the Readme file. Data files are located in the data folder, and supporting code can be found in the util folder, including the experimental (exptdata.mat) time-resolved isotropic and anisotropic scattering signals (in Q-space), and their corresponding pair-density difference signals (in real space). Simulated (simdata.mat) time-resolved scattering signals and real-space pair-density signals derived from trajectory-based simulations under different dipole assumptions. Simulated trajectory pair distances (sim_all_traj_avg.mat) including histograms of charge density distributions for specific atom pairs, averaged over multiple trajectories. Trajectory statistics (Trajset.mat) with the estimated dissociation times, velocities, and CO rotation periods for each simulated trajectory, with pair distance crossing data. Normal mode analysis (nma.mat) detailing the pair distance modulations for each normal mode, their Fourier spectra, and associated atomic displacements over time, and code demonstrating how to extrapolate trajectories using a single trajectory data (TRAJ046.mat).
