# Supplementary material for: Real-space observation of the dissociation of a transition metal complex and its concurrent energy redistribution
Source: Nat Commun. 2025 May 22;16:4767. doi: 10.1038/s41467-025-60009-8 (PMC12098838; doi:10.1038/s41467-025-60009-8)
Supplement: Supplementary file 3 — Supplementary Data 1 [file 41467_2025_60009_MOESM3_ESM.zip › FeCO5-dataset-main/feco5dataset.zip!/feco5dataset/~WRL1589.tmp]

exptdata is a matlab struct of the experimental data with fields:

t: [1×70 double] % pump-probe time delay vector (in ps)

q: [1×1225 double] % q vector (in A^-1)

R: [260×1 double] % Real space vector (in A)

S0: [1225×70 double] % Expt. Isotropic difference scattering array (q x t)

S2: [1225×70 double] % Expt. Anisotropic difference scattering array (q x t)

PD0: [260×70 double] % Expt. Pair density difference array (R x t) obtained from inverting S0

Note that the vectors t and q are not uniform.

simdata is a matlab struct of the simulated data with fields:

t: [1×741 double] % time vector (in fs)

q: [1×73 double] % q vector (in A^-1)

R: [1×520 double] % Real space vector (in A)

S0traj: [73×741 double] % Sim. Isotropic scattering array obtained from averaging all trajectories

S2atraj: [73×741 double] % Sim. Anisotropic scattering array assuming axial dipole direction

S2etraj: [73×741 double] % Sim. Anisotropic scattering array assuming equatorial dipole direction

PD0traj: [520×741 double] Sim. Pair density array (R x t) obtained from inverting S0traj

sim_all_traj_avg is a matlab struct of the simulated pair distances data with fields:

t: [1×741 double] % time vector (in fs)

Rab_grid: [1×120 double] % Real space vector (in A)

u_pairs_str: [5×2 string] % the labels of atom pair, e.g. ["C" "C"] for the first row

pairs: [120×741×5 double] % the histogram of atom pairs charge density in e^2 units, as a function of time, space, and pair type.

trajset is a matlab struct of the simulated trajectories fitted parameters with fields:

t_diss: [1×116 double] % The estimated dissociation time (in fs)

v_diss_com: [1×116 double] % The estimated dissociation velocity (in A/fs)

TCOrot: [1×116 double] % the estimated rotation period of the dissociating CO (in fs)

t_cut: [1×116 double] % the termination times of each trajectory (in fs)

GT: [1×116 logical] % the trajectories that were selected for further extrapolation, that had a termination times of at least 210 fs and a minimum of 120 fs between dissociation onset and termination.

pair_crossing: [1×1 struct] with the following fields:

RTW: [150×30 double] % a histogram of the position and times where pair distances of trajectories crossed one another (<0.1 A dist.) weighted by their pair density.

t_grid: [1×30 double] % time grid for the RTW histogram

R_grid: [1×150 double] % Real space grid for the RTQ histogram

cont_range: [20 30 40 50 60 70] % the contour line ranges for the RTW histogram (used in Fig 4c) representing pair density in e^2
